# Characterization of an Invertebrate-Type Dopamine Receptor of the American Cockroach, *Periplaneta americana*

**DOI:** 10.3390/ijms15010629

**Published:** 2014-01-06

**Authors:** Britta Troppmann, Sabine Balfanz, Christian Krach, Arnd Baumann, Wolfgang Blenau

**Affiliations:** 1 Institute of Biochemistry and Biology, University of Potsdam, Potsdam D-14476, Germany; E-Mails: b.troppmann@web.de (B.T.); Christian.Krach@gmx.de (C.K.); 2 Institute of Complex Systems (ICS-4), Forschungszentrum Jülich, Jülich D-52425, Germany; E-Mails: s.balfanz@fz-juelich.de (S.B.); a.baumann@fz-juelich.de (A.B.); 3 Institut für Bienenkunde (Polytechnische Gesellschaft), Goethe University Frankfurt, Oberursel D-61440, Germany

**Keywords:** biogenic amine, cellular signaling, dopamine, G-protein-coupled receptor, insect, salivary gland

## Abstract

We have isolated a cDNA coding for a putative invertebrate-type dopamine receptor (*Peadop2*) from *P. americana* brain by using a PCR-based strategy. The mRNA is present in samples from brain and salivary glands. We analyzed the distribution of the PeaDOP2 receptor protein with specific affinity-purified polyclonal antibodies. On Western blots, PeaDOP2 was detected in protein samples from brain, subesophageal ganglion, thoracic ganglia, and salivary glands. In immunocytochemical experiments, we detected PeaDOP2 in neurons with their somata being located at the anterior edge of the medulla bilaterally innervating the optic lobes and projecting to the ventro-lateral protocerebrum. In order to determine the functional and pharmacological properties of the cloned receptor, we generated a cell line constitutively expressing PeaDOP2. Activation of PeaDOP2-expressing cells with dopamine induced an increase in intracellular cAMP. In contrast, a *C*-terminally truncated splice variant of this receptor did not exhibit any functional property by itself. The molecular and pharmacological characterization of the first dopamine receptor from *P. americana* provides the basis for forthcoming studies focusing on the significance of the dopaminergic system in cockroach behavior and physiology.

## 1. Introduction

Cockroaches (Blattodea) not only belong to the oldest winged insects (Pterygota), but also represent well-established model organisms for basic research in neurobiology and physiology [1]. Because of their large size, simple animal maintenance, and their role as pest insects, *Periplaneta americana* and related species are well suited for studying the morphology, physiology, and pharmacology of the insect nervous system. Such research has led to a better understanding of circadian rhythmicity ([2,3]; for a review, see: [4]), the neural basis of escape behavior (for a review, see: [5]), and neuropeptide distribution and action (for a review, see: [6]). Because of the ready accessibility of intact mini-organs for various kinds of opto- and electro-physiological recordings, cockroaches are also used to study epithelial physiology and to investigate the cellular actions of biogenic amines ([7–10]; for reviews, see: [11,12]). In addition, various learning paradigms have been established for *P. americana* during the last few years [13–17].

The biogenic amine dopamine is the only physiologically relevant catecholamine in insects (for reviews, see: [18,19]) in which it is found in relatively large amounts in the central nervous system and in peripheral organs [20,21]. The distribution of dopaminergic nerve fibers has been immunohistochemically mapped in the brain of *P. americana* [22–24] and various other insects (e.g., *Drosophila melanogaster*: [25]; *Apis mellifera*: [26,27]; *Schistocerca gregaria*: [28]). Dopaminergic fibers also innervate the salivary gland complex in locusts and cockroaches ([29,30]; for a review, see: [31]). Various insect behaviors are modulated by dopamine. In *D. melanogaster*, for example, genetic and pharmacological analyses have demonstrated the importance of dopamine in sleep, arousal, light perception, circadian entrainment, feeding, and aversive conditioning [32–40]. In addition, dopamine might contribute to the regulation of larval motor function in *D. melanogaster* [41,42]. Furthermore, dopamine is involved in the regulation of exocrine [7,8,43,44] and endocrine [45,46] secretion.

Most dopamine receptors belong to the superfamily of G-protein-coupled receptors (GPCRs). In mammals, two subfamilies of dopamine receptors have been distinguished by their pharmacological properties and intracellular signaling pathways: D1-like (D_1_ and D_5_) and D2-like (D_2_, D_3_, and D_4_) receptors (for reviews, see: [47,48]). D1-like receptors activate adenylyl cyclase, whereas members of the D2-subfamily either inhibit adenylyl cyclase or couple to different intracellular second messenger systems. D1-like (=DOP1) and D2-like (=DOP3) receptors have also been characterized in *D. melanogaster* [49,50], *A. mellifera* [51,52], and other insect species. In addition, a third subfamily of dopamine receptors (=DOP2) is present in insects [53–56]. Similar to D1-like receptors, these “invertebrate type dopamine-receptors” (INDRs; for a review, see: [19]) also activate adenylyl cyclase. Phylogenetically, however, they are more closely related to α-adrenergic-like octopamine receptors expressed in insects. The classification system for insect octopamine receptors into α-adrenergic-like octopamine receptors, β-adrenergic-like octopamine receptors and tyramine (or octopamine/tyramine) receptors was introduced based on their similarities in structure and in signaling properties with mammalian adrenergic receptors [57]. INDRs and α-adrenergic-like octopamine receptors also seem to couple not only to cAMP, but also to Ca^2+^ signaling, and to share pharmacological properties [58].

In cockroaches, information has been accumulated on the pharmacological properties of dopamine receptors in salivary glands and other tissues (for reviews, see: [12,21]). In contrast, comparatively little is known about the exact repertoire and molecular properties of aminergic receptors in *P. americana* [59–61], and, until this study, no molecular data on dopamine receptors have been available. Here, we show that the mRNA encoding an INDR, which we term PeaDOP2, is expressed in the brain and salivary glands of *P. americana*. Immunohistochemical analysis has shown that the receptor protein is present in specific brain neurons with their somas being located at the anterior edge of the medulla. When stably expressed in human embryonic kidney (HEK) 293 cells, PeaDOP2 promotes the formation of cAMP with an *EC*_50_ of 160 nM for dopamine. The effect of dopamine is mimicked by (±)-2-amino-6,7-dihydroxy-1,2,3,4-tetrahydronaphthalene (6,7-ADTN) and (±)-6-Chloro-7,8-dihydroxy-3-allyl-1-phenyl-2,3,4,5-tetrahydro-1*H*-3-benzazepine (6-chloro-APB) and abolished by co-incubation with known dopamine receptor antagonists, e.g., chlorpromazine and *cis*(*Z*)-flupentixol. This study marks the first comprehensive molecular, pharmacological, and functional characterization of a dopamine receptor in the cockroach and furthers the understanding of biogenic amine signaling in this model insect.

## 2. Results

### 2.1. Molecular Cloning and Sequence Analysis of a Dopamine Receptor from *P. americana*

Using degenerate oligonucleotide primers for highly conserved GPCR transmembrane (TM) regions 6 and 7, we amplified a cDNA fragment of 109 bp from *P. americana* brain cDNA coding for a putative dopamine receptor. Rapid amplification of cDNA ends (RACE) was undertaken with gene-specific primers in order to obtain the missing 5′ and 3′ parts of the putative *Peadop2* cDNA (see Experimental Section). The full-length cDNA consists of 1815 nucleotides (*Peadop2A*; Accession No.: HG794355) and was independently amplified as a complete fragment by using two gene-specific primers. The longest open reading frame encodes a protein of 512 amino acids (PeaDOP2A) with a predicted molecular mass of 57.7 kDa. In addition, we identified a truncated splice variant of *Peadop2A*, which we named *Peadop2B* and which possessed an alternative *C*-terminal region. The nucleotide sequence of *Peadop2B* is 1470 bp in length (Accession No.: HG794356), and the longest open reading frame encodes a protein of 456 amino acids (51.1 kDa).

**Figure 1.**
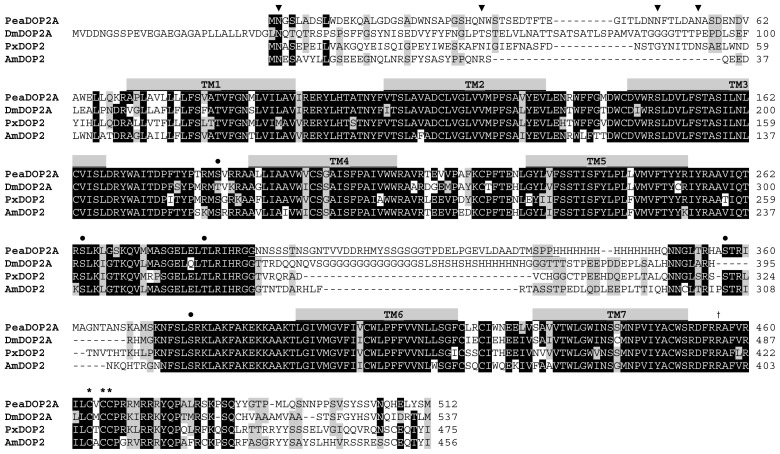
Amino acid sequence alignment of PeaDOP2A and orthologous receptors from *Drosophila melanogaster* (DmDOP2A, NP_733299), *Papilio xuthus* (PxDOP2, BAD72870), and *Apis mellifera* (AmDOP2, AAM19330). Identical residues (≥75%) are shown as white letters against black, whereas conservatively substituted residues are shaded. Putative transmembrane domains (TM1–TM7) are indicated by gray bars. Potential N-glycosylation sites (▼), PKC phosphorylation sites (●), and putative palmitoylation sites (*) of PeaDOP2A are indicated. Underlined letters represent the region within the 3rd cytoplasmic loop from which the PeaDOP2-specific antigen was derived. R_456_ (^†^) is the last amino acid residue present in the truncated variant PeaDOP2B. The amino acid position is given on the right.

Analyses of the deduced amino acid sequences of both putative receptor variants with the topology predictor *Phobius* [62] revealed the expected hallmarks of GPCRs including structural features such as seven hydrophobic transmembrane domains, an extracellular *N*-terminus, and a cytoplasmic *C*-terminal region (Figure 1). Several highly conserved sequence motifs and amino acid residues among the aminergic GPCRs are found in the PeaDOP2 receptor variants. The aspartate residue D^3.32^ according to the nomenclature of Ballesteros and Weinstein [63] in TM3 (D_151_ in PeaDOP2) is predicted to be relevant for binding the amine group of catecholamines such as dopamine, whereas serine residues in TM5 form hydrogen bonds with the hydroxyl groups of dopamine [64,65]. The characteristic C^6.47^-W^6.48^-x-P^6.50^-F^6.51^-F^6.52^ motif in TM6 (C_404_WLPFF), which interacts with the aromatic ring of dopamine, is also present. The PeaDOP2A/B receptors possess the D^3.49^-R^3.50^-Y^3.51^ (D_168_RY) sequence located at the end of TM3 involved in receptor activation (for a review, see: [19]). Furthermore, the conserved N^7.49^-P^7.50^-x-x-Y^7.53^ (N_443_PxxY) motif in TM7, which is crucial for stabilizing the inactive conformation of the receptor, is present in both variants. Biogenic amine receptors are known to have numerous post-translational modifications. The *N*-terminal region of PeaDOP2A/B contains four consensus sites for *N*-linked glycosylation (N_2_GS, N_32_WS, N_49_FT, N_55_AS; Figure 1). One consensus site for phosphorylation by protein kinase C ([S/T]-x-[R/K]) occurs in CPL2 (S_184_VR) and four additional sites in CPL3 (S_264_LK, T_282_LR, S_357_TR and S_378_RK). The *C*-terminus of PeaDOP2A harbors three cysteine residues (C_463_VCC), which are highly conserved among INDRs and serve as potential targets for palmitoylation. In contrast, the short *C*-terminal region of PeaDOP2B does not contain any cysteine residues.

BLAST searches (http://blast.ncbi.nlm.nih.gov/Blast.cgi) with the PeaDOP2A sequence indicated that it shares pronounced homology with other invertebrate-type dopamine receptors (INDRs, for a review, see: [19]). High amino acid identity (ID)/similarity (S) is shared with receptors from *Papilio xuthus* (PxDOP2; ID 63%, S 71%; [66]), *Apis mellifera* (AmDOP2; ID 62%, S 69%; [55,56]), *Ctenocephalides felis* (CfDOP2A; ID 60%, S 71%; [67]), and *Drosophila melanogaster* (DmDOP2A; ID 58%, S 66%; [53,54]). These data reflect the high conservation level of dopamine receptors and especially of receptors of the INDR class (Figure 1).

**Figure 2.**
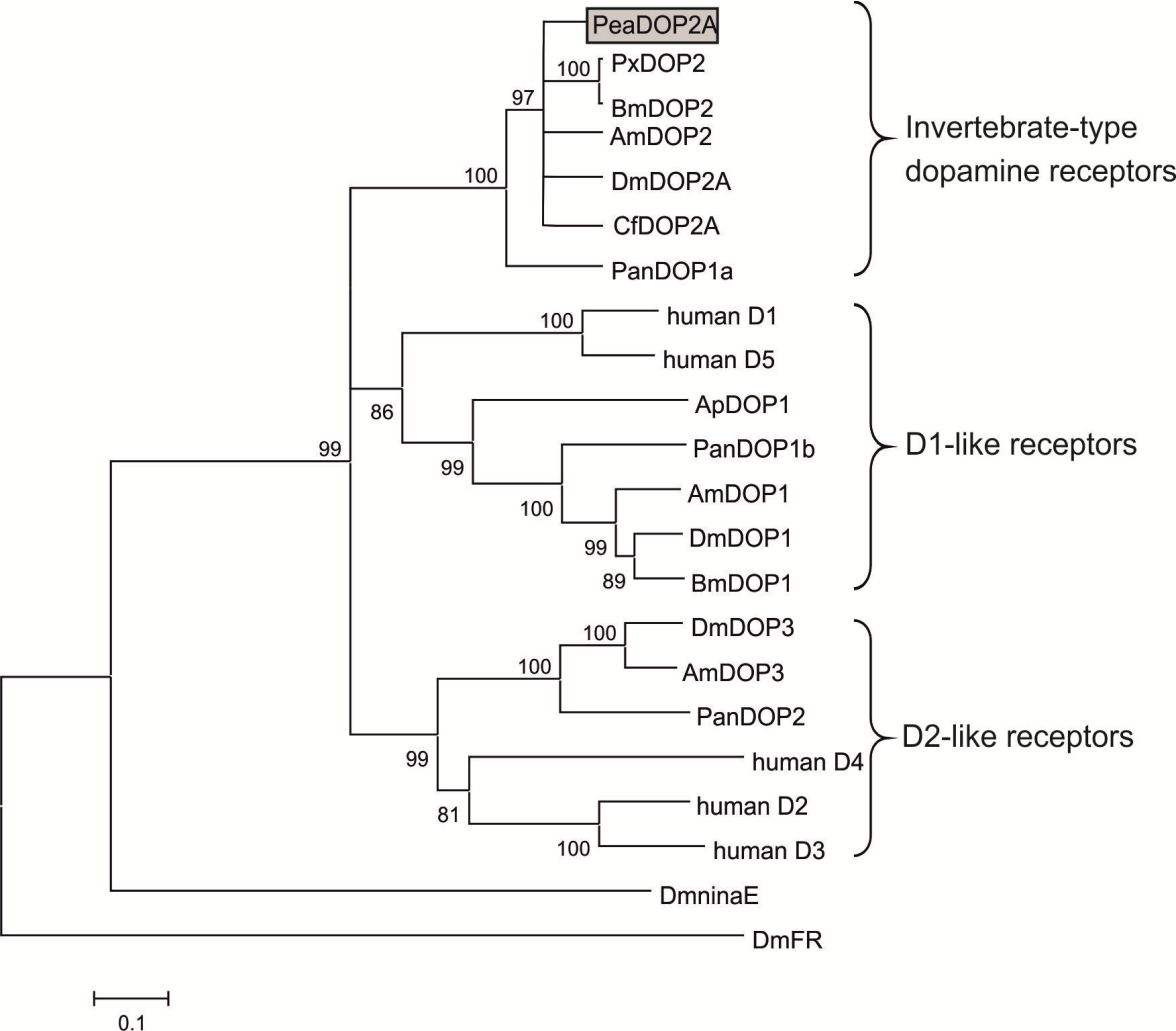
Phylogenetic analysis of PeaDOP2A and various dopamine receptors. Alignments were performed with BioEdit (version 7.0.5) by using the core amino acid sequences lacking the variable regions of the amino and carboxy terminus and the 3rd cytoplasmic loop. The genetic distance was calculated with MEGA4. The receptor sequences, followed by their accession numbers, are listed in the order illustrated: *Periplaneta americana* PeaDOP2A (HG794356), *Papilio xuthus* PxDOP2 (BAD72870), *Bombyx mori* BmDOP2 (AB162716), *Apis mellifera* AmDOP2 (AAM19330), *Drosophila melanogaster* DmDOP2A (NP_733299), *Ctenocephalides felis* CfDOP2A (DQ459405), *Panulirus interruptus* PanDOP1a (DQ295790), human D_1_ (NP_000785), human D_5_ (NP_000789), *Aplysia californica* ApDOP1 (AY918891), PanDOP1b (DQ295791), AmDOP1 (CAA73841), DmDOP1 (AAA85716), BmDOP1 (AB362162), DmDOP3 (AAN15955), AmDOP3 (AY921573), PanDOP2 (DQ900655), human D_4_ (NP_000788), human D_2_ (NP_000786), human D_3_ (NP_000787), *D. melanogaster ninaE*-encoded rhodopsin 1 DmninaE (NM_079683), and *D. melanogaster* FMRFamide receptor DmFR (AAF47700).

A multiple amino acid sequence comparison within the conserved transmembrane domains and short linker regions of PeaDOP2A in invertebrate and human dopamine receptors was used to calculate a phylogenetic tree (Figure 2). The PeaDOP2A receptor clustered with orthologous receptors of several insects and was incorporated into the branch of the INDRs.

### 2.2. Tissue Distribution of *Peadop2A* and *Peadop2B* mRNA

**Figure 3.**
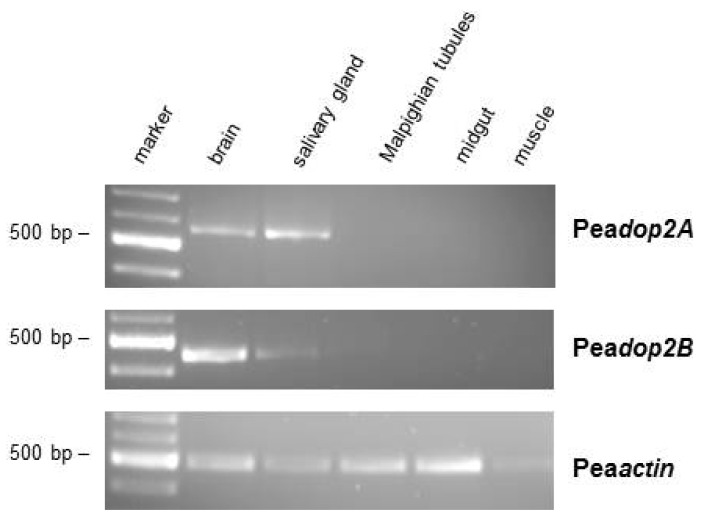
Tissue distribution of *Peadop2A/B* mRNA. A 100 bp DNA ladder (marker) is shown on the left. Detection of PCR products amplified on total RNA isolated from brain, salivary glands, Malpighian tubules, midgut, and leg muscle. Amplification failed when samples were digested with an RNase cocktail prior to RT-PCR (data not shown). The lower panel shows RT-PCR products amplified with actin-specific (Accession No. AY116670) primers as a control.

The expression pattern of *Peadop2A* and *Peadop2B* mRNA in various tissues of *P. americana* was investigated by RT-PCR with specific primers corresponding to sequences within the differing *C*-termini. Transcripts of *Peadop2A* and *Peadop2B* were detected in samples of the brain and salivary glands (Figure 3). Conversely, no receptor mRNA expression was detected in samples of Malpighian tubules, midgut, and leg muscle (Figure 3). To ensure that the fragments were not amplified from genomic DNA, samples were treated with DNase I. When tissue samples were treated additionally with an RNase cocktail, no PCR product could be amplified (data not shown).

### 2.3. Generation of an Anti-PeaDOP2 Antibody and Immunohistochemical Localization of PeaDOP2A/B Receptors

**Figure 4.**
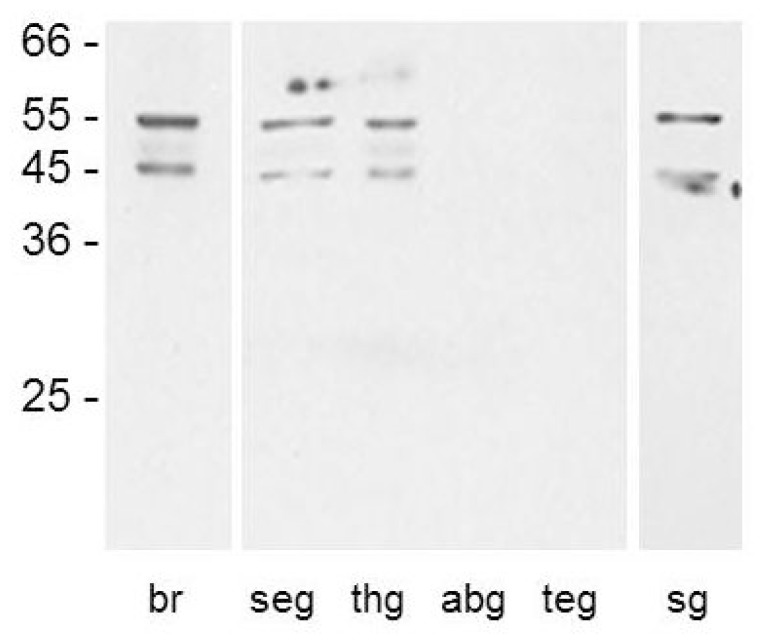
Western blot analysis with the anti-PeaDOP2 receptor antibody. Molecular weight marker in kilodalton. The anti-PeaDOP2-antibody (1:8000) recognized two bands of ~55 and ~46 kDa on Western blots containing membrane proteins (10 μg per lane) from brain tissue (br), subesophageal ganglion (seg), thoracic ganglia (thg), and salivary glands (sg). No bands were detected in samples from abdominal ganglia (abg) and the terminal ganglion (teg).

We generated a polyclonal antiserum directed against a part of the third cytoplasmic loop of PeaDOP2A/B receptors. Western blots of membrane proteins isolated from *P. americana* brain, subesophageal ganglion, thoracic ganglia, and salivary glands showed that the antibody recognized two bands of ~55 and ~46 kDa (Figure 4). These molecular weights are in accordance with the predicted molecular weights of 57.7 and 51.1 kDa for PeaDOP2A and PeaDOP2B, respectively. No bands were detected in protein samples isolated from the abdominal ganglia and the terminal ganglion (Figure 4). After pre-absorption with the peptide (15 μg/mL) used for immunization, the signal was completely lost (data not shown) supporting the specificity of the antibody for the PeaDOP2A and PeaDOP2B proteins.

**Figure 5.**
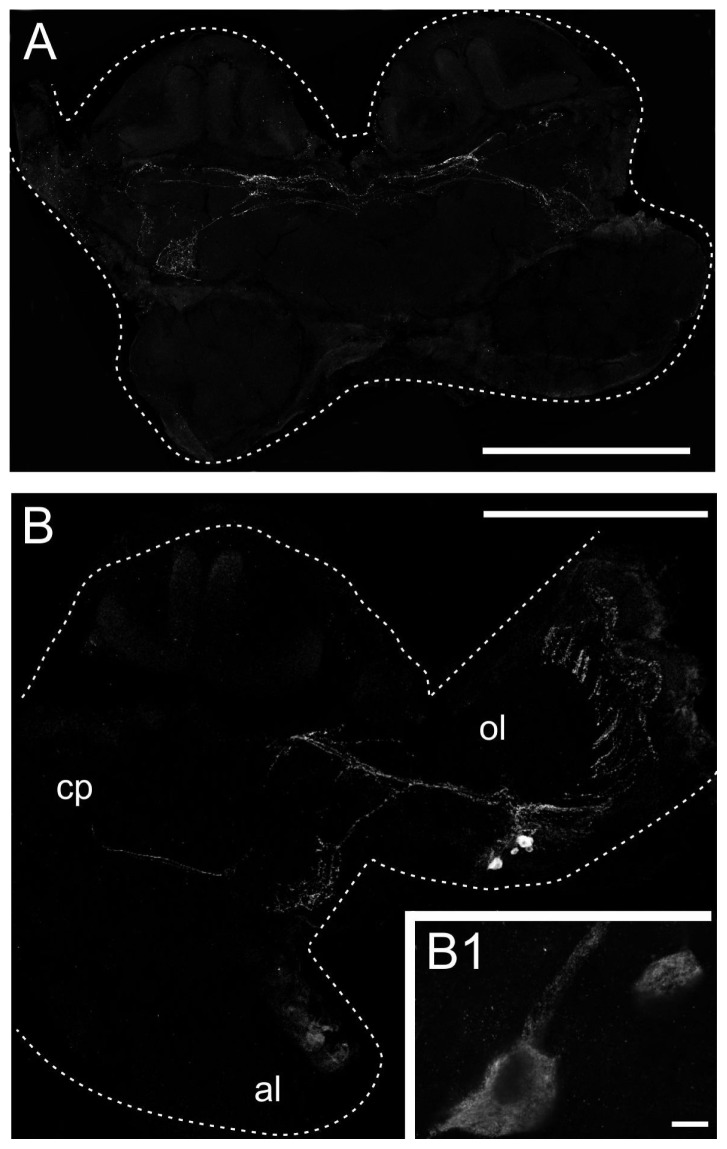
Immunohistochemical analysis of *P. americana* brain sections with the anti-PeaDOP2-antibody. Cryosections (20 μm) of the brain were labeled with primary antibody (anti-PeaDop2, 1:500). Secondary antibody was Cy2-conjugated goat anti-rabbit IgG (1:100). Images represent projections of image stacks and correspond to 10 μm. The outer margins of the sections are labeled with a stippled line. (**A**) Networks of fibers in the central protocerebrum are labeled in frontal sections of the anterior brain; (**B**) Somata and fibers in the optic lobe (ol) are labeled, in addition to fibers in the central protocerebrum (cp), in frontal sections of the central brain. al, antennal lobe. An enlargement is shown in the inset (**B1**). Scale bar in (**A**) and (**B**) is 500 μm, scale bar in (**B1**) is 10 μm.

To investigate the cellular distribution of the PeaDOP2 receptors, cryosections of *P. americana* brains were examined with these antibodies (Figure 5). In frontal sections of anterior brain regions, specific labeling was found in a network of fibers in the ventral protocerebrum and in commissures running ventrally to the mushroom bodies (Figure 5A). In sections of central brain parts, prominent labeling also occurred in large parts of the optic lobes. A group of PeaDOP2-immunoreactive somata was located ventral to the medulla. These neurons sent projections radially via the medulla (not shown) to the lamina (Figure 5B). Additional fibers projected to the ventral protocerebrum and the optic commissure (Figure 5A,B).

### 2.4. Functional Analyses of PeaDOP2A/B Receptors in HEK 293 Cells

**Figure 6.**
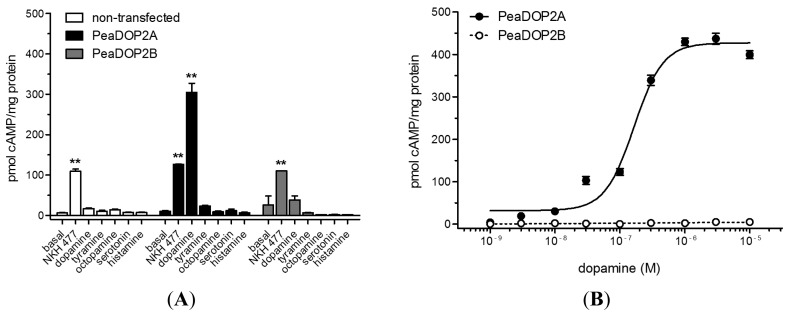

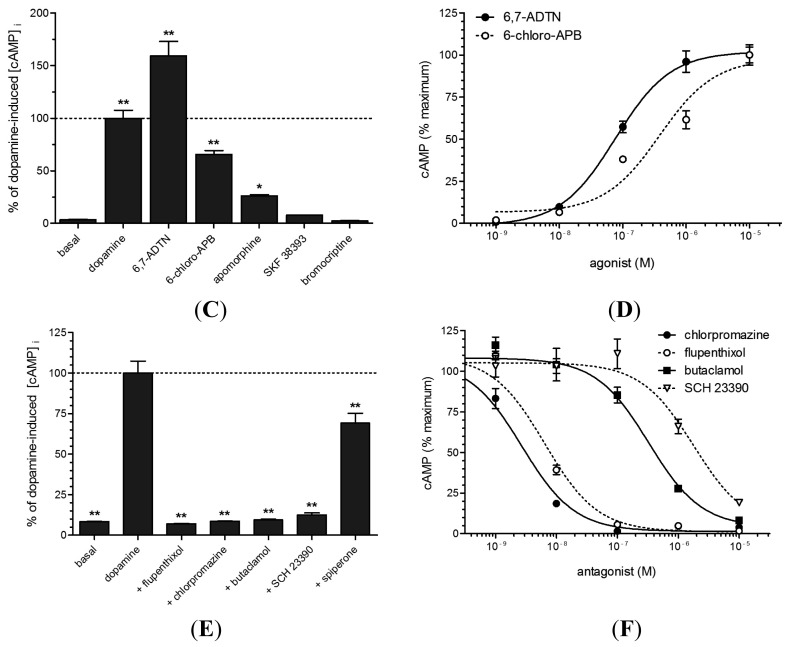
Modulation of intracellular cAMP levels [cAMP]*_i_* in HEK 293 cells stably expressing PeaDOP2A or PeaDOP2B receptors and in non-transfected cells. Data represent the mean of four to six values, and error bars indicate SEM and are, in some cases, too small to be seen. The statistical analysis is based on a one-way ANOVA followed by Dunnett’s multiple comparison test; * *p* < 0.05, ** *p* < 0.01. (**A**) Effect of the water-soluble forskolin analogue NKH 477 (an agonist of membrane-bound adenylyl cyclases) and various biogenic amines (10 μM) on [cAMP]*_i_* in non-transfected cells and in PeaDOP2A- or PeaDOP2B-expressing cells. The amount of cAMP is given as pmol cAMP/mg protein. To determine the basal [cAMP]*_i_*, cells were incubated with 100 μM IBMX only (basal). Asterisks indicate statistically significant differences for basal [cAMP]*_i_* *versus* drug-induced [cAMP]*_i_* for a given cell line; (**B**) Dose-dependent effect of dopamine (10^−9^ to 10^−5^ M) on [cAMP]*_i_* of PeaDOP2A- or PeaDOP2B-expressing cells; (**C**) Effects of dopamine receptor agonists (10 μM) on cAMP production in PeaDOP2A-expressing cells. The amount of cAMP is given as a percentage of the value achieved by stimulation with 10 μM dopamine (=100%); (**D**) Dose-dependent effects of the highly potent PeaDOP2A receptor agonists 6,7-ADTN and 6-chloro-APB (10^−9^ to 10^−5^ M). The amount of cAMP is given as a percentage of the value achieved with 10 μM 6,7-ADTN or 6-chloro-APB (=100%); (**E**) Effects of dopamine receptor antagonists (10 μM) in the presence of 300 nM dopamine on cAMP production in PeaDOP2A-expressing cells. The amount of cAMP is given as a percentage of the value achieved with 300 nM dopamine (=100%); (**F**) PeaDOP2A-expressing cells were incubated with increasing concentrations (10^−9^ to 10^−5^ M) of chlorpromazine, flupentixol, butaclamol, and SCH 23390 in the presence of 300 nM dopamine. Relative fluorescence (%) is normalized to the values obtained with 300 nM dopamine in the absence of antagonists (=100%). Of the antagonists shown, chlorpromazine displays the highest potency at PeaDop2A (*IC*_50_ = 2.7 nM).

HEK 293 cell lines constitutively expressing PeaDOP2A or PeaDOP2B receptors were generated in order to examine their second messenger coupling and pharmacological properties. Expression of PeaDOP2A/B was confirmed by Western blotting and immunocytochemistry. The ligand specificity of the receptors was tested by the application of various biogenic amines (serotonin, dopamine, tyramine, octopamine, and histamine; 10 μM). Only dopamine significantly stimulated cAMP production in PeaDOP2A-expressing cells (300 pmol cAMP/mg protein; Figure 6A). None of the biogenic amines induced a change of the intracellular cAMP level ([cAMP]*_i_*) in the cell line stably expressing the PeaDOP2B receptor (Figure 6A). In PeaDOP2A-expressing cells, the dose-dependent effect of dopamine on [cAMP]*_i_* was analyzed with concentrations ranging from 1 nM to 10 μM (Figure 6B). The dopamine effect was concentration-dependent and saturable, resulting in a sigmoidal dose-response curve (Figure 6B). Half-maximal stimulation of cAMP production (*EC*_50_) was achieved at a dopamine concentration of 160 nM (log*EC*_50_ = −6.78 ± 0.04, mean ± SEM). Maximal stimulation of cAMP synthesis was achieved at dopamine concentrations of ≥1 μM. None of the dopamine concentrations showed an effect on [cAMP]*_i_* in PeaDOP2B-expressing cells (Figure 6B) or in non-transfected cells.

Of the antagonists tested (10 μM in the presence of 300 nM dopamine), the non-selective dopamine receptor antagonists flupentixol (7.0%) and butaclamol (9.4%), the D_2_-receptor-antagonist chlorpromazine (8.6%), and the D1-receptor antagonist SCH 23390 (12.5%) inhibited the dopamine-induced increase (100%) of [cAMP]*_i_* almost to basal levels (8.6%; Figure 6E). Full dose-response curves were recorded for chlorpromazine, flupentixol, butaclamol, and SCH 23390 (Figure 6F), and *IC*_50_ values are given in Table 1.

## 3. Discussion

Aminergic receptors constitute a subfamily of rhodopsin-like GPCRs. The biogenic amine dopamine is found in deuterostomes and in protostomes and regulates and modulates numerous physiological functions and behaviors. Until now, three aminergic receptors have been cloned and experimentally examined from *P. americana*: an octopamine receptor [59], a tyramine receptor [60], and a serotonin receptor [61]. No dopamine receptor has been molecularly characterized to date. Here, we have cloned the cDNA encoding a dopamine receptor from *P. americana*, pharmacologically characterized the receptor, and investigated its expression profile.

### 3.1. Receptor Variants Occur by Alternative Splicing of the *Peadop2* Gene

Splice variants have been described for a series of aminergic GPCRs, including dopamine receptors from *Caenorhabditis elegans* [68], the crustacean *Panulirus interruptus* [69], and the insect *D. melanogaster* [50]. Splice variants differ in their tissue distribution, ligand-binding properties, G-protein coupling, and activation of signaling pathways (for a review, see: [70]). Ono and Yoshikawa [66] have shown that the genomic structure of INDRs is highly conserved within the coding region. A large part of the receptor protein ranging from the *N*-terminus, up to and including TM7, is encoded by exon 1, whereas the *C*-terminal region is encoded by exons 2 and 3. The PeaDOP2 variants identified in our study also belong to the INDR group of dopamine receptors (see below). Interestingly, a potential splice site is present at the site of divergence of the two isoforms. The *Peadop2B* variant most likely arises by retention of the intron following exon 1; this leads to an in-frame stop codon. Likewise, variants that possess truncated *C*-termini have been described in the orthologous receptor of the cat flea *C. felis* [67]. The two variants of the DOP2 receptor from *D. melanogaster* [53,54] also differ in their *C*-terminal sequence. Here, the difference arises in exon 3. Unfortunately, functional effects of the truncated *C*-terminus in *C. felis* or the differing *C*-termini in *D. melanogaster* DOP2 receptors have not been comparatively analyzed. Nevertheless, the high conservation of splice sites is in favor of a common ancestor of the INDR genes and also argues for high selection pressure retaining the functional variability by alternatively spliced INDR proteins.

### 3.2. Structural Characteristics of the PeaDOP2 Receptor

Based on bioinformatics, PeaDOP2 clearly belongs to the class of INDRs according to the nomenclature of Mustard *et al.* [19] (Figure 2). This dopamine receptor class is more closely related to α-adrenergic-like octopamine receptors than to mammalian D1-like receptors [19,58]. Hauser *et al.* [71] have postulated that, during the evolution of aminergic GPCRs, new receptors evolved that needed new ligands. Because of structural constraints, one way in which to gain such ligands was to “borrow” them from related systems. This caused so-called “ligand-hops” between receptor families. Since INDRs are not only present in hemimetabolous (this work) and holometabolous [53–56,66,67,72] insects, but also in crustaceans [73], arachnids [74], and nematodes [68], this receptor class seems to be common for the Ecdysozoa group of protostome animals. Interestingly, the cephalochordate amphioxus (*Branchiostoma floridae*), which represents the most basal of the chordates, also expresses a receptor, AmphiAmR2, showing close structural and pharmacological similarities to the INDRs [75].

The amino acid sequence of PeaDOP2 displays characteristic properties of amine-activated GPCRs in general and INDRs in particular [19]. The *N*-terminal region of the PeaDOP2 receptor contains four consensus sites for *N*-linked glycosylation (Figure 1). At least one of these sites is glycosylated when the protein is heterologously expressed in HEK 293 cells as monitored by PNGase F treatment and Western blotting (data not shown).

PeaDOP2 contains four consensus sites for phosphorylation by protein kinase C. Thus, phosphorylation of PeaDOP2 can potentially lead to the uncoupling of the receptor from G-protein-dependent signaling pathways and/or the activation of G-protein-independent pathways [76]. The *C*-terminal region of PeaDOP2A contains three cysteine residues that allow palmitoylation and, thus, formation of a fourth CPL. Palmitoylation has previously been demonstrated for several human dopamine receptors [77–79]. The high degree of conservation of these cysteine residues in INDR sequences (Figure 1) argues in favor of their functional significance for, e.g., transport to the cell membrane and localization in lipid microdomains [77,80], optimal G-protein interaction [81,82], desensitization, and internalization processes [83], and possibly also receptor oligomerization [84]. In PeaDOP2B, these cysteine residues are lacking.

Amino acid residues that are essential for dopamine binding are conserved in PeaDOP2. An aspartic acid in TM3 (D^3.32^; D_151_ in PeaDOP2) acts as a counter-ion for the protonated amino group of dopamine. Serine in TM5 forms hydrogen bonds with the catechol hydroxyl groups [64]. A tryptophan (W_405_) and a phenylalanine (F_409_) residue within the sequence motif C^6.47^-W^6.48^-x-P^6.50^-F^6.51^-F^6.52^ in TM6 interact with the aromatic ring of the ligand. This interaction modulates the bend angle of TM6 around the highly conserved proline kink at Pro^6.50^, leading to a rotation or tilting movement of the cytoplasmic end of TM6 upon activation (for a review, see: [85]). The D^3.49^-R^3.50^-Y^3.51^ sequence at the start of CPL2, which is believed to play a key role in receptor activation (for a review, see: [85]), is also present in PeaDOP2 (D_168_RY). The signature motif N^7.49^-P^7.50^-x-x-Y^7.53^ in TM7 of GPCRs is additionally present in PeaDOP2 (N_443_PVIY). This motif is crucial for internalization, sequestration, ligand affinity, stabilization of the active conformation and thus receptor activation, and receptor signaling as revealed by numerous mutagenesis studies [86–89].

### 3.3. PeaDOP2A but Not PeaDOP2B Is a Functional Dopamine Receptor

Activation of PeaDOP2A with dopamine leads to an increase in [cAMP]*_i_*, whereas tyramine, octopamine, serotonin, and histamine have no effect on [cAMP]*_i_*. The *EC*_50_ value of 160 nM for the dopamine-induced cAMP increase is similar to that of the *D. melanogaster* DOP2 receptor (350 nM, [54]). Most other insect DOP2 receptors have higher *EC*_50_ values (*A. mellifera*: 2.2 μM, [56]; *C. felis*: 2.4 μM, [67]; *Bombyx mori*: 1.3 μM, [90]). Thus, PeaDOP2A is a functional dopamine receptor with high affinity for dopamine. In contrast, dopamine stimulation of PeaDOP2B does not affect [cAMP]*_i_*. Neither PeaDOP2 receptor cause changes in [Ca^2+^]*_i_* upon dopamine incubation in the corresponding cell lines. This is in contrast to orthologous receptors from *D. melanogaster* and *A. mellifera*, which cause an increase both in [cAMP]*_i_* and in [Ca^2+^]*_i_* [53,58,91]. In summary, PeaDOP2A seems to couple exclusively to G_s_ proteins, whereas PeaDOP2B does not couple to any of the classical G-protein-mediated signaling pathways and, therefore, might be considered as a non-functional receptor.

However, our immunocytochemical analyses have shown that PeaDOP2B is translated and transported to the plasma membrane in HEK 293 cells (data not shown). Studies of various aminergic receptors suggest that truncated receptor variants might associate with full-length receptors and thereby modulate the binding and/or signal transduction properties in such heteromeric complexes. This has been shown, for example, for the D2-like receptor DOP-3 and its truncated splice variant DOP-3nf in *C. elegans* [68]. Because of an in-frame stop codon in the third intracellular loop, DOP-3nf lacks TM6 and TM7 of the full-length DOP-3 receptor [68]. DOP-3 attenuates forskolin-stimulated cAMP formation in response to dopamine stimulation, whereas DOP-3nf does not [68]. When DOP-3 was co-expressed with DOP-3nf, the ability to inhibit forskolin-stimulated cAMP formation was reduced [68]. Similar observations have been reported for human dopamine receptors and have been implicated in disease states such as schizophrenia [92–96]. Whether PeaDOP2B assembles and impairs PeaDOP2A function will be investigated in a forthcoming investigation and is beyond the scope of this study.

### 3.4. Pharmacological Properties of the PeaDOP2A Receptor

Early studies have shown that the pharmacological properties of dopamine receptors in the insect brain differ remarkably from those of their vertebrate counterparts [97,98]. This observation has been confirmed in detail for heterologously expressed dopamine receptors (for a review, see: [19]). In PeaDOP2A-expressing cells, treatments with non-selective dopamine receptor agonists (6,7-ADTN and apomorphine) and the D1-like receptor agonist 6-chloro-APB lead to an increase in [cAMP]*_i_* with a rank order of potency: 6,7-ADTN > dopamine > 6-chloro-APB > apomorphine. These agonists act similarly on INDRs from various insect species such as *D. melanogaster* (dopamine > 6-chloro-APB), *A. mellifera* (6,7-ADTN > 6-chloro-APB > dopamine > apomorphine), and *B. mori* (6,7-ADTN > dopamine > apomorphine) [53,56,90]. In contrast, the prototypical D1-like dopamine receptor partial agonist SKF 38393 does not show any effect on [cAMP]*_i_* in PeaDOP2A-expressing cells. This corresponds well to the lack of activity of SKF 38393 on all INDRs in insects [53,56,90]. The selective D2-like dopamine receptor agonist bromocriptine fails to increase [cAMP]*_i_* in PeaDOP2A-expressing cells. Since bromocriptine has been shown to be a potent agonist at insect D2-like receptors [37,50,52], it can be used pharmacologically to distinguish D2-like receptors from D1-like receptors and INDRs in insects.

The non-selective antagonists flupentixol, chlorpromazine, and butaclamol, and the selective D1-like receptor antagonist SCH 23390 are able to counteract the dopamine-induced increase in [cAMP]*_i_* fully with the following inhibitory potency: flupentixol > chlorpromazine > butaclamol > SCH 23390 >> spiperone. This antagonist profile is remarkably similar to that of orthologous receptors from, e.g., *D. melanogaster* (flupentixol > SCH 23390 > butaclamol ≥ spiperone; [53]) and *B. mori* (flupentixol > chlorpromazine > SCH 23390 > butaclamol >> spiperone; [90]). The shared pharmacological profile between PeaDOP2A with that of INDRs from other insect species further supports its classification as a functional member of the INDR class.

### 3.5. Distribution of the PeaDOP2 Receptor in the Nervous System

*Peadop2* expression in the brain was examined by RT-PCR, Western blot analysis, and immunohistochemistry. At the mRNA level, both *Peadop2A* and *Peadop2B* were detected in the brain (Figure 3). An anti-PeaDOP2-antibody was raised against a region of the CPL3 and recognized both receptor variants. On Western blots containing membrane proteins from brain, subesophageal ganglion, and thoracic ganglia, two protein bands were immunostained (Figure 4) arguing in favor of the translation of both receptor proteins, *i.e.*, PeaDOP2A/B. However, as long as no variant specific antibodies are available, it cannot be unequivocally ruled out that the occurrence of two protein bands is due to protein modification (e.g., glycosylation, degradation) or dimerization.

In *P. americana* brain sections, the anti-PeaDOP2 antibody labels a cluster of neurons in the anterior border area of the medulla, a network of fibers in the optic lobes, and fibers in the ventrolateral protocerebrum and the optic commissures (Figure 5). In the locust *Schistocerca gregaria*, the somata of 18–20 dopamine-immunoreactive tangential neurons (DTan) are clustered at the anterior edge of the medulla [28]. The somata of these neurons are clustered at the anterior edge of the medulla. Their axons project via the first optic chiasma to the posterior edge of the lamina and give rise to dense arborizations in the proximal layer of the lamina with some processes extending into the distal layer [28]. Since the DTan neurons in *S. gregaria* and the PeaDOP2-immunoreactive neurons in *P. americana* share similar distribution patterns, future double-labeling experiments with the anti-dopamine and anti-PeaDOP2 antibodies would be worthwhile. A co-distribution of the two antigens would suggest an autoreceptor function for PeaDOP2, as has been described for mammalian dopamine receptors.

The location of the PeaDOP2-immunoreactive cells between the central brain and the optic lobe is also reminiscent of the location of PDF (peptide dispersing factor)-expressing ventral lateral neurons (LNvs) described by Stengl and Homberg [99] in the cockroach *Rhyparobia maderae*. These neurons are members of the circadian clock circuitry. However, to determine whether PeaDOP2 immunoreactive cells are indeed PDF-expressing clock neurons, double-labeling experiments with anti-PDF antibodies need to be performed. Co-expression of PeaDOP2 and PDF would suggest that dopamine acting via PeaDOP2 modulates PDF-positive LNvs and thus participates in the circadian behavior of cockroaches.

The *Peadop2* expression pattern in the brain is clearly distinct from that of orthologous INDRs in the holometabolous insects *D. melanogaster*, *A. mellifera*, and *P. xuthus*, as their INDRs are predominantly expressed in the mushroom bodies of the adult brain [54,55,72].

### 3.6. Possible Function of the PeaDOP2A Receptor in Saliva Generation and Secretion

The salivary gland of *P. americana* is an established organotypic model for the investigation of the cellular actions of biogenic amines, since its secretory activity is regulated and modulated by dopamine and serotonin (for a recent review, see: [12]). Cockroaches have innervated acinar-type salivary glands with the secretory acini consisting of three cell types: peripheral cells (p-cells), central cells (c-cells), and centroacinar cells. Dopaminergic fibers project to the peripheral cells and salivary duct cells [30,100]. In isolated glands, dopamine leads to the secretion of protein-free saliva by activating the peripheral cells [7]. In addition, dopamine affects the modification of primary saliva by stimulating salivary duct cells [101]. RT-PCR and Western blotting have revealed high expression levels of PeaDOP2 in the salivary glands. The pharmacological profile of dopamine-induced salivary secretion has been investigated in detail by Marg *et al.* [8]. Notably, the order of potency for agonists (6,7-ADTN > dopamine > 6-chloro-APB >> SKF 38393 = ineffective) and antagonists (flupentixol > chlorpromazine ≥ butaclamol) is the same as for the expressed PeaDOP2A receptor. Thus, the INDR, PeaDOP2, is a likely candidate in the mediation of the effects of dopamine on salivary secretion in cockroaches, although whether a true D1-like receptor is also involved in this process remains to be examined.

In the migratory locust *Locusta migratoria*, dopamine induces hyperpolarization of salivary gland acinar cells [43] and salivary secretion [44]. The authors postulated that these effects are mediated via D1-like receptors [43,44]. Based on pharmacological experiments alone, however, it is difficult to decide whether a true D1-like receptor or an INDR mediates the effect of dopamine in the locust salivary gland. Dopamine also potently induces salivary secretion in isolated tick (*Ixodes scapularis*) salivary glands (for a recent review, see: [102]). Transcripts of a DOP1 receptor were found in salivary glands of *I. scapularis* [103]. Immunohistochemistry for the tick DOP1 receptor revealed scattered patches of reactivity in the junctions between cells on the luminal surface of specific secretory acini [102,103]. Recently, also an INDR was identified in *I. scapularis* [73] and found to be expressed in salivary glands, too [102].

## 4. Experimental Section

### 4.1. Receptor Ligands

Receptor ligands 5-hydroxytryptamine hydrochloride (5-HT), 6,7-ADTN hydrobromide, apomorphine hydrochloride hemihydrate, *S*(+)-butaclamol hydrochloride, 6-chloro-APB hydrobromide, *cis*(*Z*)-flupentixol dihydrochloride, dopamine hydrochloride, histamine dihydrochloride, (±)-octopamine hydrochloride, *R*(+)-SCH 23390 hydrochloride, *R*(+)-SKF 38393 hydrochloride, spiperone and tyramine hydrochloride were all purchased from Sigma (Taufkirchen, Germany). Chlorpromazine hydrochloride and bromocriptine mesylate were purchased from Enzo Life Sciences (Plymouth Meeting, PA, USA).

### 4.2. Cloning of *Peadop2* cDNA

Based on sequence conservation throughout various arthropod species, degenerate primers (DF1: 5′-TGYTGGBTICCITTYTT-3′; DR1: 5′-RTTDAWVYAICCIARCC-3′) were designed to amplify cDNA fragments of *P. americana* aminergic receptors [12,60,61]). Polymerase chain reaction (PCR) was performed on a *P. americana-*brain cDNA library [104] under the following conditions: 1 cycle of 2.5 min at 94 °C, followed by 35 cycles of 40 s at 94 °C, 40 s at 45 °C, and 30 s at 72 °C, and a final extension of 10 min at 72 °C. The PCR products were cloned into pGEM-T vector (Promega, Mannheim, Germany) and subsequently analyzed by DNA sequencing (AGOWA, Berlin, Germany). A specific reverse primer derived from this sequence information (SR1: 5′-ACACAAGCTCCTCGTTCCAG-3′) and an additional degenerate primer corresponding to a sequence segment of TM2 (DF2: 5′-WSIYTIGCIRTIGCIG-3′) were used for further PCR experiments. Missing 5′- and 3′-regions of the cDNA were amplified by SMART RACE (rapid amplification of cDNA ends) experiments (Clontech, Saint-Germain-en-Laye, France). Finally, PCR experiments were performed on single-stranded *P. americana-*brain cDNA to amplify the entire coding region of *Peadop2A* and *Peadop2B* by using gene-specific primers annealing in the 5′- and 3′-untranslated regions (SF: 5′-CCTAAAAAAGCGCAAGTGCGAG-3′; SRA: 5′-GGTGTGCTTACAGCGTG-3′, SRB: 5′-GTGATAAGTTTGGTTAACACTGTC-3′). The nucleotide sequences of *Peadop2A* and *Peadop2B* have been submitted to the EBI database (Accession No.: HG794355 and HG794356, respectively).

### 4.3. Multiple Sequence Alignment and Phylogenetic Analysis

Amino acid sequences used for phylogenetic analyses were identified by protein-protein BLAST searches of the NCBI database with the deduced amino acid sequence of *Peadop2A* (PeaDOP2A) as “bait”. Multiple sequence alignments of the complete amino acid sequences were performed with ClustalW. Values for identity (ID) and similarity (S) were calculated by using the BLOSUM62 substitution matrix in BioEdit 7.0.5 [105]. MEGA 4 [106] was used to calculate the genetic distances between the core sequences and to construct neighbor-joining trees with 2000-fold bootstrap re-sampling. The *D. melanogaster ninaE*-encoded rhodopsin 1 and FMRFamide receptor were used as outgroups.

### 4.4. RT-PCR Amplification of *Peadop2A* and *Peadop2B* Fragments

RT-PCR experiments to determine the tissue distribution of *Peadop2A/B* were performed as described earlier [10]. Briefly, receptor-specific fragments were amplified from 100 ng total RNA isolated from brain, salivary glands, midgut, Malpighian tubules, and leg muscle of adult male cockroaches. The sense primer was 5′-GCTGATACTATGTCTCCTC-3′ (RT-F). The antisense primers were 5′-GAGTAGAGTTCGTGTTG-3′ (RT-AR) and 5′-GGTTAACACTGTCATTC-3′ (RT-BR) for *Peadop2A* and *Peadop2B*, respectively. Amplification resulted in fragments of the expected length of 554 and 455 bp for *Peadop2A* and *Peadop2B*, respectively. RT-PCR was also performed with actin-specific primers (Accession No. AY116670) as an internal control (ActinF: 5′-CGAGTAGCTCCTGAAGAGC-3′; ActinR: 5′-GGCCTCTGGACAACGGAACC-3′; fragment length: 488 bp). cDNA was synthesized for 30 min at 50 °C followed by a denaturation step at 94 °C for 2 min. Amplification was performed for 30 cycles at 94 °C for 40 s, 60 °C for 40 s, and 72 °C for 40 s, followed by a final extension at 72 °C for 10 min.

### 4.5. Antibody Production and Purification

Antibodies were raised against a maltose-binding protein (MPB) containing a specific region of the 3rd cytoplasmic loop (CPL3) of PeaDOP2 (see Figure 1), which was constructed as follows. The CPL3-specific fragment was amplified by PCR with AB-F (5′-AAAGAATTCGCAGTAAT CCAGACG-3′) and AB-R (5′-TTTGTCGACTCAGTGATGTGGAGGAGACAT-3′) as the sense and antisense primer, respectively. The fragment was cloned into the pMAL-c2X vector (New England Biolabs, Frankfurt, Germany). The fusion protein was over-expressed in *E. coli* BL21 and purified by amylose affinity-chromatography. Anti-PeaDOP2 polyclonal antiserum was raised in rabbits (Pineda-Antikörper-Service, Berlin, Germany). For purification of crude anti-PeaDOP2-antiserum, the CPL3-specific fragment of PeaDOP2 was cloned into the pET-30a vector (Novagen, Darmstadt, Germany) and over-expressed. The purified protein was coupled to a HiTrap NHS-activated High Performance column (GE Healthcare, Freiburg, Germany). Antibodies from 50 mL crude antiserum were affinity purified as described previously [60].

### 4.6. Western Blot Analysis

Cockroach brains were homogenized in 150 μL Roti-load sample buffer (Roth, Karlsruhe, Germany) and incubated for 5 min at 95 °C. Alternatively, membrane proteins were isolated as described previously [61] and incubated for 5 min at 60 °C. Proteins were separated by 12% SDS-polyacrylamide gel electrophoresis (SDS-PAGE). Approximately 10 μg protein, as determined by a modified Bradford assay (Roti-Nanoquant, Roth, Karlsruhe, Germany), was run per lane and then transferred to polyvinylidene fluoride membranes (PVDF, Roth, Karlsruhe, Germany). PVDF membranes were labeled with antibodies as described previously [61] by applying anti-PeaDOP2 (1:8000) followed by incubation with a secondary antibody conjugated to horseradish peroxidase (1:20,000; American Qualex, La Mirada, CA, USA). For controls, antibodies were pre-absorbed with the fusion protein (15 μg/mL) used for immunization. Immunoreactivity was detected by enhanced chemifluorescence.

### 4.7. Immunofluorescence Staining of Brain Sections

Dissected brains were fixed for 2 h in 3% paraformaldehyde, 0.075 M lysine HCl, 0.01 M sodium periodate, 0.2 M sucrose in 0.1 M phosphate buffer (pH 7.0). For immunofluorescence staining, preparations were rinsed in phosphate-buffered saline (PBS), transferred to 10% (*w*/*v*) sucrose in PBS for 1 h, incubated overnight in 25% sucrose in PBS at 4 °C, and frozen in melting isopentane (−155 °C). Sections (20 μm) were cut at −30 °C on a cryostat and collected on poly-L-lysine-coated cover-slips. The sections were successively incubated in 0.01% Tween 20 in PBS, 50 mM NH_4_Cl in PBS, PBS, and blocking solution containing 1% normal goat serum, 0.8% bovine serum albumin (BSA), and 0.5% Triton X-100 in PBS, with each step lasting 5 min at room temperature. Sections were then incubated overnight at 4 °C with anti-PeaDOP2 (1:800) diluted in blocking solution, washed for 3 × 10 min in PBS, and incubated with secondary antibodies for 1 h at room temperature. Sections were washed again for 3 × 10 min in PBS and mounted in Mowiol 4.88 (Hoechst, Frankfurt, Germany) containing 2% n-propyl gallate. Fluorescent images were recorded with a Zeiss LSM 510 confocal laser-scanning microscope (Carl Zeiss, Jena, Germany).

### 4.8. Construction of pc*Peadop2A/B*-HA Expression Vector

Expression-ready constructs of *Peadop2A* and *Peadop2B* cDNA were generated by PCR. To monitor transfection efficiency and receptor protein expression, a hemagglutinin (HA) epitope tag was engineered to the 3′ end of each cDNA. First-round PCR was performed with a sense primer 5′-GATTAAGCTTCCACCATGAACGGAAGCCTAGCAG-3′ and the antisense primers 5′-TGGGACGTCGTATGGGTACATCGAGTAGAGTTCGTGTTG-3′ (for *Peadop2A*) or 5′-TGGGACGTCGTATGGGTACCTTCTGAAATCTCGACTCC-3′ (for *Peadop2B*). In second-round PCR experiments, the same sense primer was used in combination with the antisense primer 5′-TTTGGATCCTTAAGCGTAGTCTGGGACGTCGTATGGG-3′. PCR products were digested with *Bam* HI and *Hin*d III and sub-cloned into pcDNA3.1(+) vector (Invitrogen, Karlsruhe, Germany) yielding pc*Peadop2A*-HA and pc*Peadop2B*-HA. The correct insertion was confirmed by DNA sequencing.

### 4.9. Functional Expression of the PeaDOP2A/B-HA Receptor

Approximately 8 μg pc*Peadop2A/B*-HA vector was separately introduced into exponentially growing HEK 293 cells (~4 × 10^5^ cells per 5 cm Petri dish; Greiner, Frickenhausen, Germany) by a modified calcium phosphate method [107]. Stably transfected cells were selected in the presence of the antibiotic G418 at 0.8 mg/mL. Isolated foci were propagated and analyzed for the expression of PeaDOP2A/B-HA by immunocytochemistry and Western blot analysis with a commercial anti-HA antibody (anti-HA high affinity; Roche, Penzberg, Germany).

### 4.10. Functional Characterization of PeaDOP2A and PeaDOP2B Receptors

Assays to determine the ability of PeaDOP2A/B-HA receptors to stimulate adenylyl cyclase activity were performed as described earlier [108]. PeaDOP2A/B-expressing cells were grown in minimal essential medium with GlutaMAX™ (Invitrogen, Karlsruhe, Germany), 10% (*v*/*v*) fetal bovine serum, 1% (*v*/*v*) non-essential amino acids, and antibiotics (all from Invitrogen, Karlsruhe, Germany). Cells were incubated with ligands for 30 min at 37 °C in the presence of the phosphodiesterase inhibitor isobutylmethylxanthine (IBMX; Sigma, Taufkirchen, Germany; final concentration 100 μM) and lysed by adding 0.5 mL ice-cold ethanol. After 1 h at 4 °C, the lysate was transferred to a reaction tube and lyophilized. The amount of cAMP produced was determined by using the TRK 432 cyclic AMP assay kit (GE Healthcare, Freiburg, Germany). Data were analyzed and displayed by using PRISM 4.01 software (GraphPad, San Diego, CA, USA).

## 5. Conclusions

Cockroaches are established model organisms for studying circadian rhythms [4], insect learning [16], and the aminergic control and modulation of salivary secretion [12]. The pharmacological characterization and tissue localization of the first dopamine receptor provides the basis for forthcoming studies examining its role in cockroach behavior and physiology. Interference with PeaDOP2 expression by applying the RNAi technique or receptor activation with specific PeaDOP2 ligands can be used to unravel its contribution to rhythmic behavioral patterns and memory formation in this insect.

## Figures and Tables

**Table 1.** Agonist (*EC*_50_) and antagonist (*IC*_50_) profile of heterologously expressed PeaDOP2A receptor. For each substance, *EC*_50_ and *IC*_50_ values were derived from dose-response curves and are listed in order of their potency in increasing [cAMP]*_i_* and blocking dopamine (300 nM)-induced cAMP increase, respectively.

Substance	*EC*_50_/*IC*_50_ (nM)	log*EC*_50_/log *IC*_50_
**agonists**		
dopamine	163.3	−6.787
6,7-ADTN	76.4	−7.117
6-chloro-APB	381.9	−6.418
**antagonists**		
chlorpromazine	2.7	−8.569
flupentixol	6.5	−8.189
butaclamol	326.0	−6.487
SCH 23390	1786.0	−5.748
